# Be known, be available, be mutual: a qualitative ethical analysis of social values in rural palliative care

**DOI:** 10.1186/1472-6939-12-19

**Published:** 2011-09-28

**Authors:** Barbara Pesut, Joan L Bottorff, Carole A Robinson

**Affiliations:** 1 School of Nursing, University of British Columbia Okanagan, 3333 University Way, Kelowna, BC, V1V 1V7, Canada; 2 Institute for Healthy Living and Chronic Disease Prevention, University of British Columbia Okanagan, 3333 University Way, Kelowna, BC, V1V 1V7, Canada

**Keywords:** palliative care, qualitative research, ethnography, ethics, rural health services

## Abstract

**Background:**

Although attention to healthcare ethics in rural areas has increased, specific focus on rural palliative care is still largely under-studied and under-theorized. The purpose of this study was to gain a deeper understanding of the values informing good palliative care from rural individuals' perspectives.

**Methods:**

We conducted a qualitative ethnographic study in four rural communities in Western Canada. Each community had a population of 10, 000 or less and was located at least a three hour travelling distance by car from a specialist palliative care treatment centre. Data were collected over a 2-year period and included 95 interviews, 51 days of field work and 74 hours of direct participant observation where the researchers accompanied rural healthcare providers. Data were analyzed inductively to identify the most prevalent thematic values, and then coded using NVivo.

**Results:**

This study illuminated the core values of knowing and being known, being present and available, and community and mutuality that provide the foundation for ethically good rural palliative care. These values were congruent across the study communities and across the stakeholders involved in rural palliative care. Although these were highly prized values, each came with a corresponding ethical tension. Being known often resulted in a loss of privacy. Being available and present created a high degree of expectation and potential caregiver strain. The values of community and mutuality created entitlement issues, presenting daunting challenges for coordinated change.

**Conclusions:**

The values identified in this study offer the opportunity to better understand common ethical tensions that arise in rural healthcare and key differences between rural and urban palliative care. In particular, these values shed light on problematic health system and health policy changes. When initiatives violate deeply held values and hard won rural capacity to address the needs of their dying members is undermined, there are long lasting negative consequences. The social fabric of rural life is frayed. These findings offer one way to re-conceptualize healthcare decision making through consideration of critical values to support ethically good palliative care in rural settings.

## Background

Although attention to healthcare ethics in rural areas has increased [1], specific focus on rural palliative care is still largely under-studied and under-theorized [2,3]. This is a significant gap in light of an aging demographic with multiple complex chronic illnesses and economically induced migration patterns that include older adults retiring in rural areas for financial benefits and younger adults leaving rural areas because of declining employment in resource dependent industries [4]. These factors have the potential to place significant strain on rural areas that already have healthcare resource challenges.

Despite the dearth of literature addressing rural palliative healthcare ethics some issues can be extrapolated from the rural ethics literature and the palliative ethics literature. Ethical issues common to rural communities include limited economic resources; reduced health status; limited availability and accessibility of healthcare services; cultural and personal values; dual and overlapping professional-patient/family relationships; confidentiality; care-giver stress; and few resources for ethics consultations [5,6]. Layered upon these rural issues are the ethical issues inherent in delivering palliative care: communication and decision-making; advance care planning; withholding or withdrawing treatment; symptom control; and euthanasia [7-10]. Ethically good palliative care is described as honouring patient wishes to the fullest extent possible within the limits of the law [11] and as evaluating interventions in terms of their burden to benefit ratio [8]. At a macro level, palliative ethical issues surround justice and the equitable distribution of healthcare resources. It is increasingly common to see palliative care referred to as a fundamental human right [12]. According to a report of Toronto bioethicists' opinions, of the top 10 ethical challenges facing Canadians in healthcare, a shortage of rural primary care providers and teams is #4 [13]. Clearly there is a need for further exploration of healthcare ethics in rural palliative care.

The approach one takes to the study of rural healthcare ethics is important. Kelly [14] has argued that bioethics in rural health must pay attention to the lived experiences of individuals that are formed by the complex political, economic and social realities that represent the space, place and time dimensions of the rural context. It is not sufficient to use a traditional ethical approach of the autonomous subject, but rather ethical issues need to be considered within the richness of the context. Others [15] have cautioned against extrapolating urban ethical issues into rural contexts. Rather we should sensitively "go into rural areas as into a foreign land" (p. 53). This may be done best through "a kind of moral ethnography" [[16] p. 60]. This approach stands in contradistinction to the tendency to view rural places as static unitary cultures [14], or to equate rurality with being primitive or outdated [15]. Strengths such as social solidarity, close knit relationships and community commitments that hold potential for high quality, integrated healthcare [17] need to be considered as part of the ethical picture.

The purpose of this study was to conduct the type of moral ethnography described above - an exploration to gain a deeper understanding of the values informing good palliative care from rural individuals' perspectives. The study builds upon the understandings that good care is ultimately determined by the highest values which in rural areas may include self-reliance, self-care, supportive networks, a strong work ethic and a definition of health that is related to the ability to work [18]. Further, in palliative care, where quality of life is both central and highly individualized, values are best understood by watching what individuals choose in life [10]. Therefore, in this study we sought for a descriptive account of those values enacted in rural areas that represent good palliative care. Further, we wanted to know how individuals worked to enact those values and how healthcare supported or obstructed the realization of those values.

## Methods

To gain a nuanced understanding of the values informing good palliative care from rural individuals' perspective, a qualitative ethnographic approach was used in this study. This represents an important methodological advancement in biomedical ethics by using the qualitative method of ethnography to generate knowledge through which to advance ethical theory [19]. Ethnographic data provides the rich contextual background that pays attention to the lived realities against which good care must be evaluated. The study was conducted in four rural communities in Western Canada with populations of 10, 000 or less that were located three or more hours travelling distance by car from a specialist palliative care treatment centre (see Table 1). Data were collected through 95 interviews, 51 days of field work and 74 hours of direct participant observation where the researchers accompanied rural healthcare providers. Data collection occurred in two cycles over a two year period. Preliminary findings were presented to all participants after the first cycle of data collection via community meetings to ensure that the findings represented participants' experiences and to help identify important areas for further data collection. Ethical approval for this study was obtained from the University and from the Health Authorities within which these communities resided.

**Table 1:** Palliative Care Support Services by Community

Community	Palliative Care Support
**#1**	Home and community care ER services available 0800-2000 Two palliative beds in long term care
**#2**	Home and community care ER services available 24 hours per day Two palliative beds in long term care Two palliative beds on acute medical unit
**#3**	Home and community care ER services available 24 hours per day Two palliative beds in long term care Acute medical unit but no designated palliative beds
**#4**	Home and community care ER services available 24 hours per day Hospice beds attached to long term care unit Acute medical unit but no designated palliative beds

Interview participants were identified through purposive and snowball sampling. Palliative care "champions" in each community helped to ensure representation of those individuals most involved in palliative care. The sample included family members (n = 25), volunteers (n = 11), nurses (n = 27), physicians (n = 5), social workers (n = 2), healthcare administrators (n = 15), an occupational therapist (n = 1), funeral directors (n = 3), a pharmacist (n = 1) and clergy (n = 5). Seventy-two interview participants (80%) were female and 18 (20%) were male. Average length of time for participants living in the community was 29 years with a range of 2 to 82 years. Seventy (80%) participants were age 46 or older. Direct participant observation was conducted primarily with nurses who worked with palliative individuals in an acute care hospital or home setting and consisted of accompanying the nurse in the work context. Field work consisted of immersion experiences designed to enable the researchers to get to know the communities better and entailed activities such as reading local papers, visiting museums and attending functions such as the hospice palliative care society meetings.

Interviews were conducted face to face in the communities by the principal investigator or research assistant using a semi-structured interview guide. Questions solicited participants' ideas of good palliative care and the strengths, gaps, and aspirations for palliative care in their community. Participants were asked to relate stories of both positive and negative palliative care experiences. The interview guide was pilot tested on two participants and then refined. Interviews lasted from 30 to 90 minutes, were audio-taped, transcribed verbatim, checked for accuracy and entered into NVIVO qualitative software for analysis. Field notes were written of interview, observational and fieldwork experiences. These were not integrated as part of the thematic analysis but rather helped to heighten theoretical sensitivity, to inform and triangulate the findings from the interviews and to contribute to a rich contextual understanding that assisted analysis.

Questions that guided the analysis of the data were "What does this data tell us about the highest values in the context of palliative care in rural areas? What behaviours do individuals enact to ensure these values are fulfilled? How does healthcare support or obstruct these values?" The investigative team began by independently reading several transcripts and inductively identifying the most prevalent thematic values. Once these values were identified the interview transcripts were coded using NVIVO. Analytic quality measures included confirming with participants, continuing to analyze and compare coding among investigators and searching for data pieces that might contradict the findings. Participant observation and field work also played an important role in enabling understanding of how those values were enacted in the context of the community, and in particular in the context of the healthcare community.

## Results

Three primary values were constructed from an analysis of the interviews, fieldwork and participant observation: knowing and being known, being available and present, and maintaining a spirit of community and mutuality. These values were congruent across the communities within the study and across the stakeholders involved in rural palliative care. Although these were highly prized values, each came with a corresponding challenge. Being known often resulted in a loss of privacy. Being available and present created a high degree of expectation and potential caregiver strain. The values of community and mutuality created entitlement issues, presenting daunting challenges for coordinated change.

### Knowing and Being Known

Knowing and being known was the value that participants spoke of most frequently. Being known and knowing others within the community provided benefits that often countered deficits in resources. Although there are few formal dedicated palliative services in rural communities, patient needs were often known and met through an informal system of care. For example, when patients located outside of the town needed medication or a home support worker, healthcare providers often called upon neighbours they knew who might be available to fill in the gaps. Knowing provided an important level of accountability to ensure that others would not just be available, but trusted, when they were needed. As the following quote from a healthcare administrator suggests, knowing was not just valued, it was necessary for building better care.

*There really is value in getting to know the people that you work with on this team. You know, the discharge planning nurse borrows our chairs for her daughter's wedding and, you know, we see each other...And I think it's not just nice, it's essential. If you don't know these people, you're not able to develop the trust and, you know, just keep working on the commitment to make it better *(A-4).

Family members spoke of the value of being known by healthcare providers. This contrasts sharply with the lack of knowing they experienced so acutely when they had to commute to an urban centre for care by strangers. One family care provider reflected on the difference in her mother's care when she was located in an urban versus a rural center highlighting the importance of the social knowing in rural areas. The value of being known is exemplified in the following quote, which shows how both mother and daughter worked to establish important but missing connections, such as with a social worker from their home community whom the daughter could trust to know what might be best for her.

*Even just getting to know the maintenance people, the cleaning people, she became attached to [them]. It was just more of a better connection with staff I think....A social worker in the [urban] hospital got to know me because she was from [my community] originally....so that was kind of neat. She knew my situation, I don't think I got any special care or anything but she understood the best situation for myself (F-6)*.

Healthcare providers spoke of knowing the patient and family well enough to predict needs that might arise in a context where palliative care coverage was not available 24 hours per day. There was also a sense of privilege in caring for those that they knew and of having the distinction of being someone in the community who could care as illustrated by the following quote by one homecare nurse. *"Like they love doing home care nursing. They are caring for their neighbours, their colleagues, friends" *(N- 3). This knowing was also important between healthcare providers. Nurses often found themselves working on the margins of their scopes of practice, bending the rules or having to implement creative solutions to address resource shortages for their palliative patients. In this situation it was the trust and knowing between long term colleagues that allowed nurses to do their job well. Similarly, physicians were better able to enact their role when they could comfortably delegate much of the care to nurses that they knew and trusted. This long term knowing and working together as a team also helped to build palliative care capacity. In one of the study communities a core group of physicians and nurses dedicated to palliative care had worked together for over a decade. This type of stable teamwork helped to ensure that newcomers were quickly socialized into the palliative care philosophy. "*As they've hired new people to come in, it's just expected that you will embrace the team's way of doing things*" (A-1).

Individuals who were well known and had contributed to the life of the community over time had somewhat privileged positions when they became recipients of care. Participants who were prominent in the community were more likely to be extremely satisfied with the care they received than individuals who were less visible. Healthcare providers had a particularly privileged position as expressed by one nurse. *"I shouldn't say it but they (healthcare providers) get really good care. Cause if it's someone you know, let's face it I'm sorry, it's not preferential, but there's gotta be some perks for having given so much in your career to people that are going through the same thing, so we tend to go over and above. And we're happy to do it" *(N-15).

This knowing within the community also had disadvantages, the most important being the loss of privacy and anonymity. Participants spoke of the challenges of delivering and receiving intimate palliative care to those who they knew and worked with in the community and the importance of being sensitive to those difficulties. *"It's very personal too, if somebody is doing some kind of personal care on you. It's different when your neighbour is coming in and cleaning you and bathing you and doing those sorts of things that normally [in an urban centre] I've never seen you before*" (A-1). This participant went on to describe the sensitive, diplomatic work that it took to ensure individuals received the care they required when this loss of privacy would keep them from seeking help.

The loss of anonymity was also a significant drawback. Illness trajectories and grief were public events lived openly before the community. Contact with healthcare providers was often visible as individuals would meet friends and neighbours in the emergency department or would see homecare nurses coming to the door. In one situation, the death of a family member became publicly known before the family was made aware. In another situation, a grieving family member was reluctant to leave her home for weeks after the death of her husband because she could not face her grief publicly. This lack of anonymity was difficult for healthcare providers as well.

*So yea, it is a challenge, ... sometimes you'd like to be anonymous, you would just like to not walk down the street and see your chemo patients or whatever, you would rather just be able to walk down the street and not know, or you know, not know what they're going through. And it's hard when you're in a grocery line up and you see the wife of somebody that has just died, or the husband of somebody who's just died, in a big city, it's not gonna happen. But here it happens all the time, and what do I say? How are you doing? You're in a grocery line up, you know like you feel like you gotta say something but, you're both gonna end up crying in the grocery line up *(O-1).

Participants had strategies for protecting the privacy of individuals within healthcare as expressed by this participant. *"I've lived here all my life and I know a lot of people in the community and when I do come in and I see them there [in hospital], I try not to know them, because you know, really, nobody wants to know that I'm here or somebody else is there*" (A-9). The anonymous grieving available in urban communities cannot be maintained in a rural community where many know your circumstances.

### Being Available and present

The sense of knowing and being known that was so important in these rural communities inevitably led to an expectation that those you know and who know you would be available and present for you in your time of need. In general, healthcare providers valued being available and present and took pride in meeting that expectation although it came with a high cost. Many healthcare providers spoke of being on call 24 hours per day even though it was not recognized as part of their official role. Nurses provided their personal cell phone numbers to families in the last few days of life, although this was not limited to nurses as indicated by one anecdote from a nurse. "*Well if anything happens, you know in the night, I'm just going to give you my home phone okay. But don't tell anybody. And the guy laughed because five people had already done that*" (N-9). Nurses went out at night, and outside working hours, to put in catheters or pronounce death, funeral directors had family members come to their remote fishing cabins to make arrangements for their loved ones, and physicians "dropped by" on their way to holidays to check on patients living in remote areas. Even when healthcare providers moved outside of the community there was an expectation that they maintain involvement. In one community there had been a long term physician involved in palliative care, and even though he was now practicing in a remote community, nurses still contacted him for support. Family members spoke of the importance of this availability. Note that in the following quote the nurse informed the family member that she was available 24 hours per day even though this was outside of her employment responsibilities and reimbursement.

*There's no way I could find any way to improve it [palliative care]. Right off the bat [the nurse] informed us that she was available 24 hours a day. All we had to do was phone her. Our family doctor phoned, and he was available to come...so like everything that we needed in terms of patient care was there, just as close as a phone call, so I can't think of any way that you could improve that *(F-5).

Although this family member appreciated that help was just a phone call away, participants also spoke of the importance of healthcare providers being physically present. A phone call was often not enough. In these communities there were two telephone resources available, a palliative hot line for healthcare providers and a nurse line for family care providers. The palliative hot line was limited by the inability of the physician on the other end to provide orders. Likewise, family care providers needed on-site presence rather than distant support. In the words of one administrator "*They often need somebody who can go to the home and be present and make tangible their support*" (A-10). In one community there was a "quick response" nurse who was physically available to patients and families at home in the late afternoon and evening. Even a brief participant observation with this nurse illustrated the importance of this role in family support to solve problems and prevent hospital admissions. Unfortunately, budget for the position was cut during the study period. The nurse in this position had extensive palliative expertise and so a scarce and valuable human resource was lost through the cut.

This availability of healthcare providers was also somewhat problematic. Although participants suggested that it could lead to a high degree of satisfaction in their work, it could also lead to caregiver strain. For some, there was little choice about whether or not to be available. Rural palliative care has an inherent accountability that may not be present in urban areas. In urban areas many healthcare providers care for strangers - there is no ongoing obligation. However, participants in this study acknowledged that their choices had consequences in the long term. Neighbours and friends would remember their willingness, or lack thereof, to be available. One nurse described the following situation.

*One of my physician friends is actually the god-father of my daughter. [He] called me up and said, 'I've got a palliative patient who I know her, and I know her family, they go to the same church as I do, and she is dying of a nasty cancer, she's really dehydrated and we need to give her some fluid. Are you able to come and help me start the IV?' *(N-1).

This nurse went on to speak of the dilemma she felt being called at home by a friend to help with something that was outside of her legal role. Nurses in particular struggled with not knowing their professional and legal boundaries when they provided nursing care outside of the boundaries of the employer/employee relationship. They often found themselves providing care outside of their allowable hours or geographic distance and were uncertain how to document care that should not have occurred. But as one nurse expressed, "*People know each other...how could you not?*" (N-2).

Participants also recognized that this value of being available and present was not necessarily universally shared and so might not be sustainable. Older participants in the study suggested the volunteerism upon which this type of availability rested was diminishing in the younger generation. Younger physicians and nurses, or those who had been recruited from other countries to fill shortages, may be less likely to do home visits and be available 24 hours per day to palliative patients and families. One physician in particular spoke of how a patient's ability to stay at home rests primarily upon the willingness of nurses to make themselves available off hours. "*The new nurses either aren't willing to make themselves available or don't understand the usefulness of making themselves that available and so it's actually much harder to look after patients in the community*" (P-1). The consequences in this case were that many more patients were being admitted to hospital within a few hours of death. This diminishing sense of volunteerism in rural communities was cited by numerous participants across the data. Participants pondered the effect on rural capacity for palliative care in light of this diminishing willingness to be available 24 hours per day.

### Community and Mutuality

Although urban legends typically highlight the independent rural spirit, in this study there was a strong emphasis on the values of community and mutuality. As one Director of a Hospice put it, "In rural areas neighbours are not just nice, they are necessary". This value of community and mutuality was evident in the tangible help provided to palliative patients and their families and in the generous commitment to the community overall that created great rural capacity for care.

The family care providers in the study in particular provided numerous examples of the ways in which members of the community provided tangible care for them. This included everything from substantial fundraising, to home renovations to accommodate care needs, to yard work and meal preparation, to cleaning up homes after a death so that family members would not have to do it. Often it was this support from the community that made family care giving possible as depicted in the following quote from a retired nurse who had difficulty providing the extra care required for her husband while he was in hospital: "*My friends found out that it was getting very hard on me and they just took over and said, 'One of us will be there for every meal to help feed him. Set him up, feed him, get him back into position afterwards.' If it wasn't for them...." (F-11)*. Indeed sometimes the outpouring of concern from the community was overwhelming and participants spoke of declining further offers of assistance. However, this value of community and mutuality was highly reciprocal. That is, those participants that had been involved in giving to the rural community also received the highest amount of support. Participants expressed concern for those individuals who were new to the community, who had not been involved in community organizations such as churches or who had been disenfranchised over the years. The same generosity expressed toward neighbours was also evident toward healthcare in general. Participants spoke of the substantial donations that came in to support hospice societies and other healthcare initiatives.

The value of community was also evident in how closely healthcare providers worked together with a view to the entire rural community. This was particularly effective when healthcare providers had their offices co-located and so much informal communication flowed seamlessly. For example, in one of our study communities the discharge planner and the homecare nurses had offices housed adjacent to the acute medical unit. And so, even when formal palliative rounds did not occur, there was a constant flow of communication between healthcare providers about palliative patients even as they faced transitions in places of care. All members of the team were aware and negotiated palliative patients' needs on a daily basis, making for high quality and seamless transitions in care. There were also rural 'champions' in these communities who had worked over decades on behalf of the community to build capacity for palliative care. It was largely these champions, rather than the health authorities, that contributed to the sustainability of any sort of palliative program over time.

However, this value of community and mutuality also meant that participants generally had a strong sense of ownership over healthcare in their community. Unpopular policies or changes initiated by the health authority often met resistance. Indeed, there was a real tension between having the community raise substantial funds that made new initiatives possible but then having to turn the administration of those initiatives over to health authority decision makers who were not located locally in the community. Decision making by strangers, at a distance, contravened all the values underpinning effective palliative care in these rural communities. For example, there was a highly contentious policy that palliative patients occupying long term care beds would be charged a per diem fee. Healthcare providers felt great moral distress when patients were transferred from their home or acute care into a long term palliative care bed and then 'charged to die'. Participants spoke of the covert strategies they enacted to ensure that individuals would not have to go to these beds if they chose not to. Indeed, it was ironic to see the costs of keeping palliative individuals in acute care to circumvent that per diem fee, a fee that was a nominal amount to the health authority but a substantial amount to the individuals having to pay the fee.

In summary, the extensive amount of ethnographic data collected in this study highlighted the important underlying values of being known, being available and present, and dedication to community and mutuality for the provision of good rural palliative care. These values are essential to take into account when considering healthcare change in rural communities. We would like to illustrate the impact of not taking these values into account when a change was made in one rural community that had long term negative effects on palliative care and community morale.

### A Case Study: Closure of an acute care facility

Some healthcare facilities are aging and difficult decisions need to be made about whether they should be updated and kept open. These facilities have a prominent role in the community when individuals have generously donated to them over time and many friends and loved ones have received care there. In one of our study sites a decision was made to close down the acute care facility and create a regional hospital in a community twenty minutes away. Twenty-four hour 'emergent' care was continued through the emergency department in the home community and the expectation was that the local physicians would provide their services to the regional hospital. However, after a short period of time the local physicians withdrew from participation in the new facility which created difficulties in providing adequate coverage. The solution was to hire hospitalists, physicians who 'covered' the hospital for four days at a time on a rotating basis. However, what was largely unanticipated was the effect on the local medical community, the resistance to travelling for care, and the effects of having palliative patients cared for by individuals that did not know them well.

The local hospital had been a place where physicians gathered, had coffee, and communicated vital information about individuals under care. They had immediate contact with all of the care providing team as this was the facility where all of the offices were co-located. However, once the hospital was closed these physicians no longer carried on that vital communication and medical care became largely disconnected in the community.

*So now, the difference is there is no coffee room. There is no meeting of people. There isn't much collegiality, I mean nurses have a segment of collegiality because they all work in one place and talk to each other. The modern way of giving care, it's very much more individualistic, who knows what I do anymore? It used to be so... so the discussion of problem issues whatever, there's less intermingling (P-1)*.

Patients and families were resistant to travelling to the new facility for care. It was strange to them, they did not have access to their own healthcare providers and it was often difficult to get there. Transportation can be difficult in rural communities and there is no obligation to provide public transport between communities. As some participants indicated, even though the adjacent community was only 20 minutes away, it was typically not a place they would ever visit and so to go there for care was foreign. This resulted in the unfortunate situation of palliative patients who required emergency symptom management having to travel by ambulance to a strange community for care and not necessarily having the support of family and friends or even their own physician. The complexity of triaging and solving the palliative care challenges was exacerbated by the fact that these hospitalists did not know the medical or social history of the patients. As one long-term rural physician suggested, it is knowing the patient and family well, over time that is the basis for the clinical acumen that allows physicians to anticipate and solve problems quickly and enable palliative patients to return home. In this situation, palliative patients ended up dying in a hospital in a strange community. And although for urban residents a 20 minute commute to a hospital which is strange to them may be a normal occurrence, for rural residents this situation was less than ideal.

What was most interesting in this study was that although this facility closure had occurred almost a decade prior, participants still felt the change acutely. This depth of response can best be understood by recognizing how it violates some of the highest values held by these rural residents. At their most vulnerable time, some rural residents were required to go to a strange place to die. They were not known and they did not have the support of those who did know them as they could not be present and available. This reality was even more poignant when many of these individuals had given generously to establish the network of healthcare in their own community over their lifetime and had the expectation that the care would be there when they needed it. The knowing between healthcare providers that filled in the gaps in resources in rural communities was broken, and the sense of community and rapport that comes from being co-located as a healthcare team no longer existed - further exacerbating the isolation that many rural healthcare providers experience. This case study illustrates the essential importance of social values in the analysis of what constitutes ethically good care at end of life. In this situation, what may have been a reasonable and prudent decision from a fiscal perspective ran counter to the values that were most essential to those residing in this rural community, thus leaving a long lasting negative impression about the quality of palliative care.

## Discussion

While many of the ethical challenges we have identified are not new findings, our unique contribution to rural healthcare ethics and rural palliative care is the framing of these tensions in the context of a small number of pervasive values that underpinned ethically good rural palliative care in the study communities. The *Coalition for Rural Health Care Ethics *[5] formulated an agenda for rural healthcare ethics that specifically identified ethical concerns arising from the insufficient recognition of cultural values, which this study addresses. The values framework proposed here enables a better understanding of rural culture, what constitutes good rural palliative care and why that is so. Values are clearly one of the contextual dimensions of rural culture that must be taken into account when considering ethical issues in rural palliative care [14]. The values expressed by participants in this study echo the broader Canadian social values in relation to healthcare of a collective and caring responsibility for all citizens [20]. Further, they support Wilson et al.'s [21] findings that suggested that rural persons feel they have unique perspectives on a good death that includes a deep commitment to community and those individuals dying within their community.

Although there is a dearth of literature that specifically addresses rural palliative healthcare ethics, our findings support those of others who have identified ethical tensions arising in the context of rural healthcare. The dual personal-professional relationships that form the cornerstone of knowing and being known in a rural community present challenges to relational boundaries, confidentiality, and anonymity [2,5,22-25]. The interweaving of personal and professional relationships raises confidentiality concerns, for example there is evidence that rural patients and providers worry about and may withhold documentation for fear that other healthcare professionals, who may also be friends, will have access to the information [22,24]. The value of knowing and being known helps us understand the importance placed on a generalist model of palliative care in rural settings, where palliative care is seen as a normal and desirable aspect of continuous care from birth to death [2,24,26]. Further, this value helps explain what may be rural-urban differences in attitudes toward health care professionals at end of life. Gessert [4] and his colleagues found that the nature of family/health care provider relationships differed along rural/urban lines. "Rural participants demonstrated a more accepting and sympathetic attitude toward the healthcare providers, often offering explanations about any shortcomings. Their stories reflected their strong belief that they had admitted their relatives to the care of their neighbours and friends" (p. 22). In contrast, urban families described adversarial, demanding interactions associated with admitting their relatives to the care of strangers who would not necessarily provide "the kind of care you would want to receive" (p. 23). For rural residents, the value of knowing and being known supports trusting relationships and confidence in care.

It is evident that the work associated with being present and available is challenging but generates a high degree of commitment from all involved in the provision of rural palliative care. In fact, some rural health care professionals refer to the palliative care they offer as a 'way of life'[2]. Healthcare professionals take pride in their ability to meet patient and family needs, often going to extraordinary lengths to care for patients, risking dangerous weather conditions and working beyond their allotted hours [24]. This socially oriented context of care that invites going beyond 'the call of duty' can be associated with high personal cost [27] and can lead to a sense of isolation and burnout [28]. It has been noted that there is also a high degree of family caregiver burden associated with rural palliative care [29]. So, while rewarding, being present and available is not without risks. The development and maintenance of palliative care competencies is often mentioned as a challenge for health care professionals involved in rural palliative care [30-32]. Difficulty acquiring and maintaining essential knowledge and skills has the potential to undermine providers' ability to effectively be present and available. However, there is evidence that when health care professionals are present and available to each other via such activities as mentoring and teamwork, competencies can be nurtured on the job and the experience of being emotionally as well as practically supported is sustaining [2]. Further challenges to the value of being present and available include healthcare workforce shortages, which are endemic in rural communities [33], elimination of palliative positions, and secondment of palliative clinicians to fulfill vacancies in other areas [24].

The strong value of community and mutuality that characterized life in our study communities has been noted as a strength that supports high quality, integrated health care through social solidarity, close knit relationships, and community commitment [17]. In the rural context, a spirit of cooperation helps to overcome resource challenges [26,32]. However, a trend we found toward diminishing commitment to volunteerism has also been found by others [31] and may compromise this strength over time.

What is interesting is the matter of fact way that many of our participants dealt with what have been seen as common rural ethical issues that have the potential to generate moral distress. They were simply a part of rural life and as our findings suggest, participants had creative and diplomatic ways of dealing with issues like dual relationships, confidentiality and privacy. Indeed, in some cases what might be considered an ethical issue from an urban perspective helped to fulfill the values that participants felt were most important. For example, the loss of privacy and confidentiality was offset by the value of being known and cared for by those who would have ongoing accountability within the community. Healthcare providers derived great satisfaction from playing a vital and meaningful role as part of community mutuality.

What was particularly evident in the data were system wide ethical tensions, and in particular how rural values played into broader relations of power that were made manifest through healthcare policies and restructuring. Issues such as the per diem fee attached to formal palliative care beds, the deletion of positions held by highly valued community members and the closing of a community hospital violated the values held by participants and as such became important ethical issues. Our findings strongly support Panelli et al.'s [34] argument that we need to be continually testing policy discourse against the lived experience of health services. This is essential in rural areas where policies are often generated from those residing in urban areas. Further, what this study illustrates is the need for a systems level approach to ethics, what Vernillo [35] has referred to as preventive ethics. The goal of preventive ethics is to "improve healthcare quality by identifying, prioritizing and addressing healthcare ethics at a system level" (p. 61). For rural palliative care this entails focussing on the system issues that violate deeply held rural values and seeking to provide healthcare in a way that optimizes rather than undermines rural capacity.

The most compelling ethical issues from the perspective of participants in this study were related to system wide healthcare changes that restructured important healthcare resources in their community because of the negative impact on the provision of good palliative care. This was typified by the closure of the community hospital which resulted in residents having to travel to a neighbouring community for essential care. The deletion of positions within the community that were occupied by valued community members because of system wide budget shortfalls was also highly contentious. These policy decisions undermined existing capacity within the rural community - but more importantly were often misaligned with their core values. When participants made claims like "neighbours are not just nice, they are necessary" they were making critical statements about the roles that community and mutuality play when facing challenges with limited resources, whether those challenges be health, economic or other. When those social relationships were disrupted, vulnerabilities were created that were then difficult to redress. This supports Gessert's [4] view that social contracts, those tacit understandings that bind communities together, are important for examining rural end of life care.

Other studies, and most importantly those coming out of the field of geography and health, shed important light on these findings. Castleden et al. [36] used place as an analytic tool to examine palliative care provision in rural areas of British Columbia. They found that for rural residents distance was not simply physical but rather an emotional construct imbued with social meanings and that the aesthetics of place were important. This concurs with participants in our study who were highly reluctant to travel even relatively short distances (from an urban perspective) for care if the social and emotional aesthetics of place were not there. One could argue that at the end of life, where quality of life is so essential, this issue is even more compelling. Farmer et al. [37] conducted a qualitative study of older persons' health service provision in rural Scotland. They suggested that misaligned perspectives between management and rural residents are largely a result of differing assumptions about how health and community work. Rural community members valued community based solutions and tended to see health services, social care, transport, meals and housing as all connected. Managers and policy makers on the other hand emphasized the sharing of services across communities and tended to view service delivery in silos. James' [38] compelling analysis of the closure of rural hospitals in Saskatchewan highlights these different perspectives. She concludes that although closing rural hospitals may make sense from an economic or social determinant of health standpoint (that is moving away from an emphasis on illness-care to primary prevention) such closures often fail to take into account the role that hospitals play in rural communities. Hospitals are imbued with a meaning in the community that includes issues of identity, security and economy. Even though logical alternatives are provided, these solutions fail to address the important role that the hospital plays in the overall social fabric of the community. This was illustrated well in our study where participants still acutely felt the loss of their community hospital almost a decade later. Other studies have likewise supported the importance of rural community hospitals in end of life care [39-41].

These findings are particularly relevant for initiatives that propose that palliative care in rural communities is best supported through centralized hubs of care located outside of the community [42,43]. Although this is an intriguing idea, it should be considered as an additional support to rural communities rather than a substitute for unique solutions to building capacity within the community. Evidence from this study illustrating how communication flows in a rural community, the value of knowing and being known, the intermingling of social and collegial professional relationships, the pride that community members take in their localized resources, and the reluctance to travel even short distances for care if that care is delivered by strangers suggests that the primary solutions should be found within the communities themselves. To do otherwise is to work against the very social fabric and values that characterize rural communities.

## Limitations

Although the inclusion of four rural communities and extensive field work over a period of two years adds credibility to study findings, we acknowledge that the study communities were within a single province and were all rural as opposed to remote. Hugo [44] has suggested that rurality is largely defined by a set of social living conditions whereas remoteness is largely defined by inaccessibility. Therefore, it would be difficult to extrapolate these findings to remote communities. Further, we are conscious that simply by writing about rural values we run the risk of reifying the idea that rural culture is homogenous [14]. In describing these values our intent is not to suggest that they characterize all of rural life but rather to show how they play into building rural capacity for palliative care and how initiatives that disrupt these values also disrupt care. By virtue of our snowball sampling technique we are representing the voices of those who tend to be most visible in the community be we know less about those who do not access palliative care. It is possible that they would espouse a somewhat different set of values. Indeed, participants suggested that those who are most visible and influential in rural communities have somewhat privileged positions and we know little about those who are not connected into the system.

## Conclusion

This study illuminated the core values of knowing and being known, being present and available, and community and mutuality that provide the foundation for ethically good rural palliative care. These values offer the opportunity to better understand common ethical tensions that arise in rural healthcare and key differences between rural and urban palliative care. Context is important to understanding ethical issues. In particular, these values shed light on problematic health system and health policy changes. Clearly, when initiatives violate deeply held values and hard won rural capacity to address the needs of their dying members is undermined, there are long lasting negative consequences. The social fabric of rural life is frayed. We offer one way to re-conceptualize healthcare decision making through consideration of critical values in order to support ethically good palliative care in rural settings.

## Funding

This work was supported by the Canadian Institutes of Health Research [grant number EOG 90308]. BP is supported by a Canada Research Chair (Tier 2)

## Competing interests

The authors declare that they have no competing interests.

## Authors' contributions

BP collected data, participated in analysis of the data and drafted the manuscript. JB participated in analysis of the data and assisted with drafting the manuscript. CR collected data, participated in analysis of the data and assisted with drafting the manuscript. All authors read and approved the final manuscript.

## Pre-publication history

The pre-publication history for this paper can be accessed here:

http://www.biomedcentral.com/1472-6939/12/19/prepub

## References

[B1] Nelson WA Greene MA West A Rural healthcare ethics: No longer the forgotten quarter Cambridge Quarterly of Healthcare Ethics 2010 19 510 517 10.1017/S096318011000040X 20719029

[B2] Robinson CA Pesut B Bottorff JL Mowrey A Broughton S Fyles G Rural palliative care: A comprehensive review Journal of Palliative Medicine 2009 12 3 253 258 10.1089/jpm.2008.0228 19216703

[B3] National Rural Health A Providing hospice and palliative care in rural and frontier areas 2005 Kansas City, MO: National Rural Health Association

[B4] Gessert CE Klugman CM, Dalinis PM Rural-urban differences in end-of-life care: Reflections on social contracts Ethical Issues in Rural Health Care 2008 Baltimore: John Hopkins University Press 15 33

[B5] Nelson W Pomerantz A Howard K Bushy A A proposed rural healthcare ethics agenda Journal of Medical Ethics 2007 33 136 139 10.1136/jme.2006.015966 17329381 PMC2598268

[B6] Klugman CM Dalinis PM Klugman CM, Dalinis PM Introduction Ethical Issues in Rural Health Care 2008 Baltimore: John Hopkins University Press 1 12

[B7] Doyle D Though one man's eyes: A 30-year retropective on ethical issues in palliative care 199921929779

[B8] Cimino JE A clinician's understanding of ethics in palliative care: an American perspective Critical Reviews in Oncology Hematology 2003 46 17 24 10.1016/S1040-8428(03)00004-0 12672515

[B9] Kinlaw K Ethical issues in palliative care Seminars in Oncology Nursing 2005 21 1 63 68 10.1053/j.soncn.2004.10.009 15807058

[B10] Randall F Downie RS Palliative care ethics: A companion to all specialties 1999 Oxford: Oxford University Press

[B11] National Consensus Project for Quality Palliative Care Steering Committee National consensus project for quality palliative care: Clinical practice guidelines for quality palliative care, Executive Summary Journal of Palliative Medicine 2004 7 5 611 627 15588352 10.1089/jpm.2004.7.611

[B12] Brennan F Palliative care as an international human right Journal of Pain and Symptom Management 2007 33 5 494 499 10.1016/j.jpainsymman.2007.02.022 17482036

[B13] Breslin JM MacRae SK Bell J Singer PA Top 10 health care ethics challenges facing the public: Views of Toronto bioethicists BMC Medical Ethics 2005 6 5 108 10.1186/1472-6939-6-5 PMC1180442 15978136

[B14] Kelly SE Bioethics and rural health: Theorizing place, space, and subjects Social Science and Medicine 2003 56 2277 2288 10.1016/S0277-9536(02)00227-7 12719181

[B15] Hardwig J Rural health care ethics; What assumptions and attitudes should drive the research The American Journal of Bioethics 2006 6 2 53 54 10.1080/15265160500506845 16500854

[B16] Prata Miller J Defining 'research' in rural healthcare ethics The American Journal of Bioethics 2006 6 2 59 61 10.1080/15265160500506894 16500857

[B17] Nelson W Lushkov G Pomerantz A Weeks WB Rural health care ethics: Is there a literature? The American Journal of Bioethics 2006 6 2 44 50 10.1080/15265160500506746 16500852

[B18] Nelson WA Klugman CM, Dalinis PM The challenges of rural health care Ethical Issues in Rural Health Care 2008 Baltimore: John Hopkins University Press 34 59

[B19] Singer PA Pellegrino ED Siegler M Debate: Clinical ethics revisited BMC Medical Ethics 2001 2 1 online 10.1186/1472-6939-2-1 PMC32193 11346456

[B20] Wilson DM Kerr JR An exploration of Canadian social values relative to health care American Journal of Health Behaviour 1998 22 2 120 129

[B21] Wilson DM Fillion L Thomas R Justice C Bhardwaj PP Veillette A-M The 'good' rural death: A report of an ethnographic study in Alberta, Canada Journal of palliative care 2009 25 1 21 29 19445339

[B22] Purtilo R Sorrell J The ethical dilemmas of a rural physicianthe Hastings Center Report 1986 16 4 24 28 10.2307/3563112 3744797

[B23] Roberts LW Battaglia J Epstein RS Frontier ethics: Mental health care needs and ethical dilemmas in rural communities Psychiatric Services 1999 50 4 497 503 10211730 10.1176/ps.50.4.497

[B24] Robinson CA Pesut B Bottorff JL Issues in rural palliative care: Views from the countryside Journal of Rural Health 2010 26 78 84 10.1111/j.1748-0361.2009.00268.x 20105272

[B25] Simon RI Williams IC Maintaining treatment boundaries in small communities and rural areas Psychiatric Services 1999 50 11 1440 1446 10543853 10.1176/ps.50.11.1440

[B26] O'Connor M Lee-Steere R General Practitioners' Attitudes to Palliative Care: A Western Australian Rural Perspective Journal of palliative medicine 2006 9 6 1271 1128 10.1089/jpm.2006.9.1271 17187535

[B27] Denz-Penhey H Murdoch JC A student view of the difference between general practice and rural and remote medicine Rural and Remote Health 2007 7 641 650 17477793

[B28] McConigley R Kristjanson L Morgan A Palliative care nursing in rural Western Australia International journal of palliative nursing 2000 6 2 80 90 11035627 10.12968/ijpn.2000.6.2.8948

[B29] Hughes P Ingleton MC Clark D Providing cancer and palliative care in rural areas: A review of patient and carer needs Journal of Palliative Care 2004 20 1 44 49 15132076

[B30] Evans R Stone D Elwyn G Organizing palliative care for rural populations: a systematic review of the evidence Family practice 2003 20 3 304 310 10.1093/fampra/cmg312 12738700

[B31] Kelley ML Sellick S Linkewich B Rural nonphysician providers' perspectives on palliative care services in northwestern Ontario, Canada The Journal of Rural Health: Official journal of the American Rural Health Association and the National Rural Health Care Association 2003 19 1 55 62 10.1111/j.1748-0361.2003.tb00542.x 12585775

[B32] Rosenberg JP Canning DF Palliative care by nurses in rural and remote practice The Australian Journal of Rural Health 2004 12 4 166 171 10.1111/j.1440-1854.2004.00591.x 15315546

[B33] Van Vorst RF Crane LA Barton PL Kutner JS Kallail KJ Westfall JM Barriers to quality care for dying patients in rural communities The Journal of Rural Health: Official journal of the American Rural Health Association and the National Rural Health Care Association 2006 22 3 248 253 10.1111/j.1748-0361.2006.00040.x 16824170

[B34] Panelli R Gallagher L Kearns R Access to rural health services: Research as community action and policy critique Social Science and Medicine 2006 62 1103 1114 10.1016/j.socscimed.2005.07.018 16185802

[B35] Vernillo A Preventive ethics and rural healthcare: Addressing issues on a systems level American Journal of Bioethics 2008 8 4 61 62 10.1080/15265160802147116 18576262

[B36] Castleden H Crooks VA Schuurman N Hanlon N "It's not necessarily the distance on the map ... ": Using place as an analytic tool to elucidate geographic issues central to rural palliative care Health & place 2010 16 2 284 290 10.1016/j.healthplace.2009.10.011 20005147

[B37] Farmer J Philip L King G Farrington J MacLeod M Territorial tensions: Misaligned management and community perspectives on health services for older people in rural and remote areas Health and Place 2010 16 275 283 10.1016/j.healthplace.2009.10.010 19906556

[B38] James AM Closing rural hospital in Saskatchewan: On the road to wellness? Social Science and Medicine 1999 49 1021 1034 10.1016/S0277-9536(99)00180-X 10475667

[B39] Payne S Kerr C Hawker S Seamark D Davis C Roberts H Jarrett N Roderick P Smith H Community hospitals: an under-recognized resource for palliative care Journal of the Royal Society of Medicine 2004 97 9 428 431 10.1258/jrsm.97.9.428 15340022 PMC1079584

[B40] Payne S Hawker S Kerr C Seamark D Roberts H Jarrett N Smith H Experiences of end-of-life care in community hospitals Health & Social Care in the Community 2007 15 5 494 501 10.1111/j.1365-2524.2007.00714.x 17685995

[B41] Steers J Brereton L Ingleton C Palliative care for all? A review of the evidence in community hospitals Int J Palliat Nurs 2007 13 8 392 399 18018819 10.12968/ijpn.2007.13.8.24538

[B42] Cinnamon J Schuurman N Crooks VA Assessing the suitability of host communities for secondary palliative care hubs: a location analysis model Health Place 2009 15 3 792 800 19269241 10.1016/j.healthplace.2009.01.003

[B43] Crooks VA Castleden H Schuurman N Hanlon N Visioning for secondary palliative care service hubs in rural communities: a qualitative case study from British Columbia's interior BMC Palliat Care 2009 8 15 10.1186/1472-684X-8-15 19818139 PMC2763848

[B44] Hugo G Cocklin C, Dibden J The state of rural populations Sustainability and Change in Rural Australia 2005 Sydney: University of New South Wales Press 56 79

